# Is there an association between ABO blood types and depressive symptoms among Japanese healthcare workers during the COVID-19 pandemic?

**DOI:** 10.1371/journal.pone.0256441

**Published:** 2021-08-27

**Authors:** Dong Van Hoang, Shohei Yamamoto, Takako Miki, Ami Fukunaga, Zobida Islam, Maki Konishi, Tetsuya Mizoue

**Affiliations:** Department of Epidemiology and Prevention, Center for Clinical Sciences, National Center for Global Health and Medicine, Tokyo, Japan; Kyushu University, JAPAN

## Abstract

ABO blood types could be a biological predisposition for depression. The present cross-sectional analysis was conducted amid the second wave of COVID-19 in Japan during July 2020. We wanted to investigate the association between ABO blood types and depressive symptoms among workers (352 men and 864 women, aged 21–73 years) of a medical institution in Tokyo, Japan, which took a leading role in the response to COVID-19 in the country. A Poisson regression model with a robust variance estimator was used to estimate the prevalence ratio (PR) and 95% confidence interval (CI) for depressive symptoms associated with ABO blood types. Overall, the prevalence of depressive symptoms (using two questions employed from a Two-question case-finding instrument) was 22.0%. The adjusted PRs (95% CI) for depressive symptoms, comparing the carriers of blood type O, A, and AB with those of type B, were 0.88 (0.66, 1.18), 0.81 (0.62, 1.07), and 1.07 (0.74, 1.53), respectively. There was no difference in the prevalence of depressive symptoms between non-B and B carriers. The present study did not support the association of ABO blood types with depressive symptoms.

## Introduction

Depression continues to be a worldwide public health concern which has affected more than 264 million people [1]. This health condition has become more prevalent since the occurrence of the ongoing COVID-19 pandemic [2]. Among healthcare workers who engaged in COVID-19-related work, the overall prevalence of depression was 22.8%, but was higher among doctors (25.4%) and nurses (30.3%) [3]. Among healthcare workers at a tertiary hospital in Japan, the prevalence of depression was 27.9% during the COVID-19 pandemic [4]. Identification of vulnerability factors for depression may help to establish strategies to mitigate the burden of depression, especially under the circumstance of this COVID-19 pandemic.

Biological and epidemiological studies suggest that there may be a link between ABO blood types and the vulnerability to depression. Dysregulation of the dopaminergic system may underlie the pathophysiological mechanisms of depression [5, 6]. Dopamine beta-hydroxylase (DBH) is an enzyme that converts dopamine into norepinephrine [7]. Thus, alteration in the expression of the DBH gene could affect the regulation of this system. The disequilibrium linkage between the genes encoding ABO blood types and DBH (on chromosome 9q34) [8, 9] suggests that genetic variation in the ABO blood types may indicate an alteration in DBH expression. Previous studies [10] have shown that blood type B is associated with lower DBH activity, while blood types O and A are associated with higher DBH activity, which may increase one’s vulnerability to depression [11]. This suggests that those with blood type A and O may carry a higher risk of depression compared to those with type B. However, epidemiological evidence on this issue remains inconclusive [12–15].

Therefore, we examined the association between ABO blood types and depressive symptoms among workers of a designated medical institution for COVID-19 in Tokyo, Japan.

## Methods

### Study design and participants

A cross-sectional study was conducted amid the second wave of the COVID-19 in Japan, in July 2020, among the staff members of the National Center for Global Health and Medicine (NCGM), Tokyo, Japan, which has been taking a leading role in combatting COVID-19 since the early phase of the pandemic [16]. The primary aim of the survey was to estimate the seroprevalence of SARS-COV-2 antibodies among NCGM’s staff members who have engaged in COVID-19 related tasks. Eligible participants were defined as those who had worked in COVID-19-related departments, engaged in any COVID-19-related tasks (e.g., providing medical care, conducting studies related to SARS-Cov-2 infection, cleaning rooms or items used by COVID-19 patients, or taking temperature of visitors), or who were nurses of inpatients wards. With the help of human resource department, all eligible staff members were listed and invited to the survey (n = 1,555). For a comparison purpose, some staff who did not meet the inclusion criteria were also invited (n = 24). As the present study focused on the relationship between ABO blood types and depressive symptoms, we considered all of the survey’s participants to be eligible.

Of 1,579 workers invited, 1,228 (77.8%) agreed to participate (353 men and 875 women). We excluded 12 people with missing information on ABO blood types, leaving an analytical sample of 1,216 participants. Information on health status, preventive behaviors against COVID-19 and lifestyles were collected via an online questionnaire.

The study procedure was in accordance with the 1964 Helsinki declaration and its later amendments and was approved by the NCGM Ethics Committee (approval number: NCGM-G-003598-00). Written informed consent was obtained from all participants included in the study.

### Outcome variable

Depressive symptoms were assessed using two questions, which were employed from the Two-question case-finding instrument [17]: “During the past month, have you often been bothered by feeling down, depressed, or hopeless?”; and “During the past month, have you often been bothered by little interest or pleasure in doing things?”. In that original instrument, depressive symptoms were defined when respondents answered ‘yes’ to either of the questions (sensitivity: 95%; specificity: 65%) [17]. According to a recent meta-analysis [18], that instrument was a simple and reliable tool, which has high sensitivity and low negative likelihood ratio, for the screening of depressive symptoms in primary care settings. To improve the specificity in the present study, we defined depressive symptoms when respondents answered “yes” to both questions. In a previous Japanese study which evaluated this Two-question case-finding instrument for the screening of depressive symptoms in workplace settings, the same definition (as used in our study) yielded a sensitivity of 87.9% and a specificity of 81.4% for the identification of depressive symptoms [19].

### Exposure variable and covariates

ABO blood types (A, B, AB, and O) were self-reported by the participants. The accuracy of this type of self-reported information among Japanese nurses could reach 94.7% [20].

We selected the following covariates based on the epidemiological evidence for their association with the risk of depression: body mass index (BMI) [21], smoking status [22], alcohol consumption [23, 24], leisure-time physical activity [25], comorbid chronic conditions [26], occupation [4], working hours [27], and sleep duration [28] and engagement in Covid-19 related work [29].

The information on sex, age (in years), and occupation was collected from the Labour Management Office of the NCGM. We grouped occupation into four categories: nurses, doctors, allied healthcare professionals and others. The information on the other covariates (including height and weight) was self-reported by participants. Smoking status was defined as non-current smoker or current smoker depending on the use of either traditional or heat-not-burn cigarettes. Daily consumption of alcohol was estimated based on two questions on the frequency (do not or quit drinking, 1–3 days/month, 1–2 days/week, 3–4 days/week, 5–6 days/week and everyday drinking), and amount of alcohol consumed per day (0.5, 1, 1.5, 2, 2.5, 3, 3.5 and ≥ 4 drinks). One drink is equivalent to 1 *go*, a Japanese traditional unit which contains approximately 23 g of ethanol. Alcohol consumption was then categorized into three groups (never or quit drinking, < 1 and ≥ 1 *go*/day). Leisure-time physical activity was assessed with three questions for the time per week participants spent on indoor physical activity, outdoor physical activity during daytime and outdoor physical activity during night-time. Each question had seven response options: never, < 30 min, 30–59 min, 1–1.9 h, 2–2.9 h, 3–3.9 h and ≥ 4 h per week. The total hours of these physical activities were calculated and categorized into four groups (not at all, < 1, 1–1.9 and ≥ 2 h/week). The comorbid chronic condition was defined as having any of the following diseases: diabetes, hypertension, chronic obstructive pulmonary disease, heart diseases, cerebrovascular diseases, cancer and other chronic diseases. Response options for sleeping duration in the previous 1 month were: < 4, 4–4.9, 5–5.9, 6–6.9, 7–7.9 and ≥ 8 h/day, which were grouped into three (< 6, 6–6.9 and ≥ 7 h/day). Response options for daily working hours in March to mid-April were: < 6, 7, 8, 9, 10, 11, 12, 13 and ≥ 14, which were grouped into three (≤ 8, 9–10 and ≥ 11 h/day). Engagement in Covid-19-related work referred to the involvement in any tasks which might increase the risk of contracting with the SARS-CoV-2 viruses, such as providing medical care for COVID-19 patients, cleaning rooms or items used by the patients, tasks related to biological specimens (sampling, testing or disposal), taking temperature of visitors or outpatients, and working as general receptionists. A complete list of COVID-19-related tasks is available in S1 Appendix.

### Data analysis

One-way analysis of variance (ANOVA) and Chi-squared test were respectively used to compare the distribution of continuous and categorical variables across ABO blood types. The descriptive statistics of participants’ characteristics were presented as mean (standard deviation [SD]) for continuous variables, and as percentage (%) for categorical variables. Poisson regression with robust variance estimators were used to investigate the association between ABO blood types and depressive symptoms. Two models were fitted: Model 1 was adjusted for background factors: age, sex and occupation; and Model 2 was further adjusted for other factors: BMI, smoking status, alcohol consumption, leisure-time physical activity, comorbid chronic condition, engagement in Covid-19 related work, working hours, and sleep duration. All analyses were carried out in RStudio (Version 1.3.1056) [30]. The level of significance was set at p < 0.05 (two-sided).

## Results

As shown in Table 1, the mean age was 36.1 (SD: 11.0) years. Of the 1,216 participants, 28.9% were men, 49.6% were nurses, 69.1% engaged in Covid-19 related work, 11.9% worked more than 10 hours per day, 58.6% never or spent less than 1 h per week in leisure-time physical activity, and 44.3% slept less than 6 hours per day. There was no measurable difference in the background factors according to ABO blood types (p > 0.05 for all variables).

**Table 1:** Characteristics of participants, according to ABO blood types.

Characteristics	Total, n (%)	Blood type, n (%)				P value ^‡^
		A	B	AB	O	
	n = 1,216	n = 488	n = 247	n = 118	n = 363	
**Age (year), mean (SD) **	36.1 (11.0)	36.2 (11.3)	36.0 (10.6)	36.5 (10.7)	36.0 (11.1)	0.97
**Sex (% men) **	352 (28.9)	130 (26.6)	73 (29.6)	46 (39.0)	103 (28.4)	0.07
**BMI (kg/m**^**2**^**), mean (SD)**	21.7 (3.3)	21.6 (3.3)	22.0 (3.6)	21.9 (3.7)	21.6 (2.9)	0.44
**Occupation**						
Nurse	603 (49.6)	255 (52.3)	112 (45.3)	58 (49.2)	178 (49.0)	0.51
Doctor	247 (20.3)	101 (20.7)	46 (18.6)	23 (19.5)	77 (21.2)	
Allied healthcare professional	155 (12.7)	52 (10.7)	37 (15.0)	15 (12.7)	51 (14.0)	
Other ^**†**^	211 (17.4)	80 (16.3)	52 (21.1)	22 (18.6)	57 (15.8)	
**Covid-19 related work **	840 (69.1)	338 (69.3)	171 (69.2)	78 (66.1)	253 (69.7)	0.90
**Working hours per day ** ^**††**^						
≤ 8	656 (53.9)	251 (51.4)	137 (55.5)	59 (50.0)	209 (57.6)	0.054
9–10	415 (34.2)	179 (36.7)	74 (30.0)	44 (37.3)	118 (32.5)	
≥ 11	145 (11.9)	58 (11.9)	36 (14.5)	15 (12.7)	36 (9.9)	
**Smoking status **						
Current non-smoker	1,148 (94.4)	463 (94.9)	232 (93.9)	113 (95.7)	340 (93.7)	0.77
Current smoker	68 (5.6)	25 (5.1)	15 (6.1)	5 (4.3)	23 (6.3)	
**Alcohol consumption (*go*/day)**						
Never or quit drinking	357 (29.3)	146 (29.9)	70 (28.3)	34 (28.8)	107 (29.5)	0.94	
< 1	717 (59.0)	281 (57.6)	150 (60.8)	68 (57.6)	218 (60.0)		
**≥ **1	142 (11.7)	61 (12.5)	27 (10.9)	16 (13.6)	38 (10.5)		
**Leisure-time physical activity (h/week)**							
Not at all	339 (27.9)	143 (29.3)	74 (30.0)	40 (33.9)	82 (22.6)	0.25	
< 1	373 (30.7)	146 (29.9)	75 (30.4)	31 (26.3)	121 (33.3)		
1–1.9	200 (16.4)	87 (17.8)	34 (13.7)	16 (13.5)	63 (17.4)		
**≥ **2	304 (25.0)	112 (23.0)	64 (25.9)	31 (26.3)	97 (26.7)		
**Sleep duration (h/day) **							
< 6	538 (44.3)	224 (45.9)	113 (45.7)	50 (42.4)	151 (41.6)	0.87	
6–6.9	489 (40.2)	191 (39.1)	99 (40.1)	47 (39.8)	152 (41.9)		
≥ 7	189 (15.5)	73 (15.0)	35 (14.2)	21 (17.8)	60 (16.5)		
**Comorbid chronic diseases **	200 (16.4)	77 (15.8)	47 (19.0)	23 (19.5)	53 (14.6)	0.38	

^**‡**^ obtained from one-way ANOVA or Chi-squared test

^**†**^ included clerical and administrative staff

^**††**^ refers to the period between March and April 2020, about three months before the survey

Overall, the prevalence of depressive symptoms was 22.0%. As shown in Table 2, there were no significant differences in the prevalence of depressive symptoms across ABO blood types. The adjusted prevalence ratio (PR) (95% confidence interval [CI]) for depressive symptoms, comparing the carriers of blood types O, A, and AB with those of type B was 0.88 (0.66, 1.18), 0.81 (0.62, 1.07), and 1.07 (0.74, 1.53), respectively. In addition, there were no significant differences in depressive symptoms between the carriers of non-B and those of B type (PR: 0.87; 95% CI: 0.68, 1.12).

**Table 2:** Association of ABO blood types with depression prevalence.

Blood type	N (%)	Depressive symptoms (%)	Prevalence ratio (95% CI)	
			Model 1	Model 2
Overall** **	1,216 (100.0)	22.0		
B** **	247 (20.3)	24.7	1.00 (ref.)	1.00 (ref.)
O** **	363 (29.9)	20.9	0.85 (0.63, 1.14)	0.88 (0.66, 1.18)
A** **	488 (40.1)	20.5	0.81 (0.62, 1.07)	0.81 (0.62, 1.07)
AB** **	118 (9.7)	25.4	1.05 (0.73, 1.53)	1.07 (0.74, 1.53)
Non-B** **	969 (79.7)	21.3	0.85 (0.67, 1.09)	0.87 (0.68, 1.12)

Non-B: carriers of blood type A, B and AB; Model 1: adjusted for age, sex and occupation (nurse, doctor, allied healthcare professional, other); Model 2: adjusted for body mass index (kg/m^2^), smoking status (current non-smoker, current smoker), alcohol consumption (never or quit drinking, < 1, ≥ 1 *go*/day), leisure-time physical activity (not at all, < 1, 1–1.9, ≥ 2 h/week), comorbid chronic condition (yes, no), engagement in Covid-19 related work (yes, no), working hours (< 6, 6–8, 9–10, ≥ 11 h/day), sleep duration (< 6, 6–6.9, ≥ 7 h/day) in addition to covariates included in the Model 1.

## Discussion

Among the 1,216 health workers of a medical institution designated for COVID-19 in Japan, depressive symptoms were not associated with ABO blood types. To the best of our knowledge, this is the first study to examine the relationship between ABO blood types and depressive symptoms among Japanese people.

Similar to the present study, a few other cross-sectional studies did not find any association between ABO blood types and depressive symptoms. Yadav et al. [15] failed to identify a significant difference in depression scores across ABO blood types among 315 Indian dental students. A recent Croatian study [14] found that the prevalence of ABO blood types were similar between patients with mood disorders and healthy controls. Another study in the US [31] also failed to observe a significant association of ABO blood types with either unipolar or bipolar disorders. In contrast, blood types A and O were associated with a higher incidence of postpartum depression among Chinese women [13], and type O was associated with higher depression scores among the US adults [12]. In an earlier report based on the secondary analysis of six hospital-based studies [31], blood type O was more prevalent among people with bipolar disorders compared with other diagnoses, although the observed difference was small and varied significantly across studies.

The null finding of the present study might be explained by the weak impact of the linkage between the genes encoding DBH and ABO blood types on the dopamine serum level [32]. According to Cowen [33], a small reduction in serum dopamine may provoke depressive symptoms in those with a history of previous depressive disorders, but may not be sufficient to trigger depressive symptoms in healthy individuals. The association between ABO blood types and depression observed in previous hospital-based studies [31] was mainly observed among psychiatric patients who were much more vulnerable to subtle changes in serum dopamine [33]. Meanwhile, participants in our study were generally healthy workers. For this reason, the link between ABO blood types and depression, if it exists, might be too weak to be revealed in the present study. In addition, our study was conducted at a medical institution where SARS-Cov-2 patients were examined and treated; the psychological impact of COVID-19 might affect all participants regardless of their blood type. Thus, this widespread impact might blur any subtle differences in the prevalence of depressive symptoms across ABO blood types.

The present study has following limitations. First, we examined the association with prevalence, but not incidence, of depressive symptoms. Thus, we were not able to infer whether ABO blood types are associated with the development of depressive symptoms. Second, the self-administered questionnaire we used for the assessment of depressive status has been validated in a Japanese population, but not among healthcare workers. Additionally, the associations of ABO blood types with depressive symptoms assessed by using a questionnaire may differ from those with clinically-diagnosed depression. Third, some participants might misreport ABO blood types, leading to an attenuation in association [34]. Nevertheless, the distribution of ABO blood types among our study participants (A: 40.1%, B: 20.3%, AB:9.7% and O:29.9%) is similar to that in other Japanese studies (A: 37.6% - 39.0%, B: 22.1% - 23.7%, AB: 8.8% - 9.8% and O: 28.6% - 30.3%) [32, 35, 36], and the self-report of ABO blood types by Japanese medical professionals is accurate (94.7%) [20]. Therefore, it is unlikely that the present null finding is ascribed to the misreporting of ABO blood types. Finally, the present finding in a medical institution may not be generalized to the general population of Japan.

In conclusion, the present cross-sectional study among Japanese hospital workers did not show any evidence to suggest a linkage of depressive symptoms to ABO blood types.

## Supporting information

S1 Appendix COVID-19-related works. (DOCX)
