# Supplementary material for: Is there an association between ABO blood types and depressive symptoms among Japanese healthcare workers during the COVID-19 pandemic?
Source: PLoS One. 2021 Aug 27;16(8):e0256441. doi: 10.1371/journal.pone.0256441 (PMC8396730; doi:10.1371/journal.pone.0256441)
Supplement: S1 Appendix — (DOCX) [file pone.0256441.s001.docx]

**S1 Appendix: COVID-19-related works**

1. Health check-up for those passengers who were on charter flights from Wuhan, China (except for patient care).
2. Health check-up for passengers on the Diamond Princess and the Costa Atlantica (except for patient care).
3. COVID-19 test spot (PCR test center), fever outpatients.
4. Work related to care facilities for mildly ill patients.
5. Work performed in close proximity to the patients (< 1m) (e.g., medical treatment, specimen collection, surgery, nursing care, equipment installation, etc.).
6. Work performed at a slight distance (1m or more) from the patients (i.e., work mentioned in the response option 5 and, reception, guidance, transportation, paperwork, surveys, etc.).
7. Test specimens (testing, research, etc.).
8. Work related to specimen but other than those described in #7 (transport, disinfection, disposal, etc.).
9. Work related to rooms, clothing and items used by patients (cleaning, washing, disinfection, disposal, etc.).
10. Temperature test for visitors, outpatients; and general reception.
